# Comparing barriers to breastfeeding success in the first month for non-overweight and overweight women

**DOI:** 10.1186/s12884-018-2094-5

**Published:** 2018-11-27

**Authors:** Kimberley M. Mallan, Lynne A. Daniels, Rebecca Byrne, Susan J. de Jersey

**Affiliations:** 1 School of Behavioural and Health Sciences, Australian Catholic University, 1100 Nudgee Rd, Banyo, QLD 4014 Australia; 2 School of Exercise and Nutrition Sciences, Queensland University of Technology, Victoria Park Rd, Kelvin Grove, QLD 4059 Australia; 3 Department of Nutrition and Dietetics, Royal Brisbane and Women’s Hospital, Brisbane, Australia

**Keywords:** Breastfeeding, Infant formula, Maternal obesity

## Abstract

**Background:**

Women who enter pregnancy overweight or obese tend to have poorer breastfeeding outcomes compared to non-overweight women. Women’s experiences of specific breastfeeding-related problems and reasons for use of formula have not been systematically investigated according to pre-pregnancy BMI. The aim of this study was to compare self-reported breastfeeding problems in non-overweight and overweight women and identify the main reasons for use of infant formula during the first month postpartum.

**Methods:**

The present study involved a cross-sectional secondary analysis of data collected as part of a hospital-based longitudinal study of women that commenced in pregnancy (~ 16 weeks). At ~ 4 months postpartum Australian women (*N* = 477) self-reported breastfeeding problems and reasons for use of infant formula during the first month postpartum. Pre-pregnancy BMI was calculated based on self-reported pre-pregnancy weight and measured height. Binary logistic regression analyses were used to compare pre-pregnancy weight status groups (“non-overweight” [BMI < 25 km/m^2^] and “overweight” [BMI ≥25 km/m^2^]) on self-reported breastfeeding problems and reasons for use of infant formula. Analyses were adjusted for covariates that differed between groups (*P* < .1).

**Results:**

Frequency of self-reported breastfeeding problems was similar across weight status groups. “Not enough milk” was the predominant reason for giving infant formula. Overweight women were more likely than non-overweight women to agree that infant formula was as good as breastmilk.

**Conclusions:**

Overall it does not appear that overweight women are more likely to experience a range of specific breastfeeding problems in the first months compared to non-overweight women. However, the severity and duration of the problems needs to be examined. Breastfeeding interventions need to addresses concerns around milk supply as these are common and are likely to be of universal benefit however overweight women in particular may benefit from guidance regarding the benefits of breastfeeding for both themselves and their infants.

## Comparing barriers to breastfeeding success in the first month for non-overweight and overweight mothers

Breastfeeding confers multiple benefits to both mother and child including reduction of child obesity risk [1, 2]. However poorer breastfeeding outcomes in terms of initiation, exclusivity and duration are associated with higher maternal body mass index [BMI]); [3, 4] a characteristic independently associated with increased child obesity risk [5]. Breastfeeding problems such as issues with latching and concerns about the infant getting enough milk have been associated with early cessation of breastfeeding, particularly during the first month postpartum [6, 7]. However, it is not clear whether overweight and obese women are necessarily more likely to experience specific breastfeeding problems than their non-overweight counterparts and whether such problems are more likely to adversely impact on exclusive (or any) breastfeeding for overweight and obese women. Thus, understanding reasons for early cessation of exclusive or any breastfeeding particularly in high risk populations (e.g., overweight or obese mothers) is a fundamental step in designing targeted interventions that can support breastfeeding success and potentially reduce risk of childhood obesity.

Reasons for overweight or obese women to cease exclusively breastfeeding have been explored in a number of qualitative studies. Birth complications (caesarean delivery), feeling self-conscious and a perception of low milk supply were cited as reasons for early cessation of exclusive or any breastfeeding [8, 9]. Although informative, these studies still do not clarify whether these reasons for cessation of breastfeeding are unique to, or more prevalent in, overweight or obese mothers compared to non-overweight mothers and thus can explain differential feeding outcomes.

In a study on breastfeeding outcomes of Australian women enrolled in the *New Beginnings: Healthy Mothers and Babies* study, [10] it was shown that significantly fewer overweight compared to non-overweight women were exclusively breastfeeding at hospital discharge (73% vs 86%) or at 4 months postpartum (50% vs 66%,) despite no difference in breastfeeding initiation between overweight and non-overweight women (98% vs 96%) [11]. The aim of the present study is to extend on this previous work using data from the *New Beginnings: Healthy Mothers and Babies* study to explore whether these differences in breastfeeding outcomes may be related to differences in self-reported breastfeeding problems and reasons for formula use between non-overweight and overweight women. Specifically, this study examined whether overweight and non-overweight women differed in their reporting of (i) a range of specific breastfeeding problems in the first month postpartum; (ii) use of infant formula in response to these specific breastfeeding problems, and (iii) important reasons for use of infant formula in the first month postpartum. Based on these aims it was predicted that (i) overweight women would report be more likely than non-overweight women to report breastfeeding problems in the first month and (ii) be more likely to use formula in response to these problems. The final aim (iii) was mostly exploratory however overweight women were predicted to endorse reasons for using infant formula related to insufficient milk supply and feeling self-conscious feeding in public more than non-overweight women.

## Methods

### Study design and participants

**Fig. 1:**
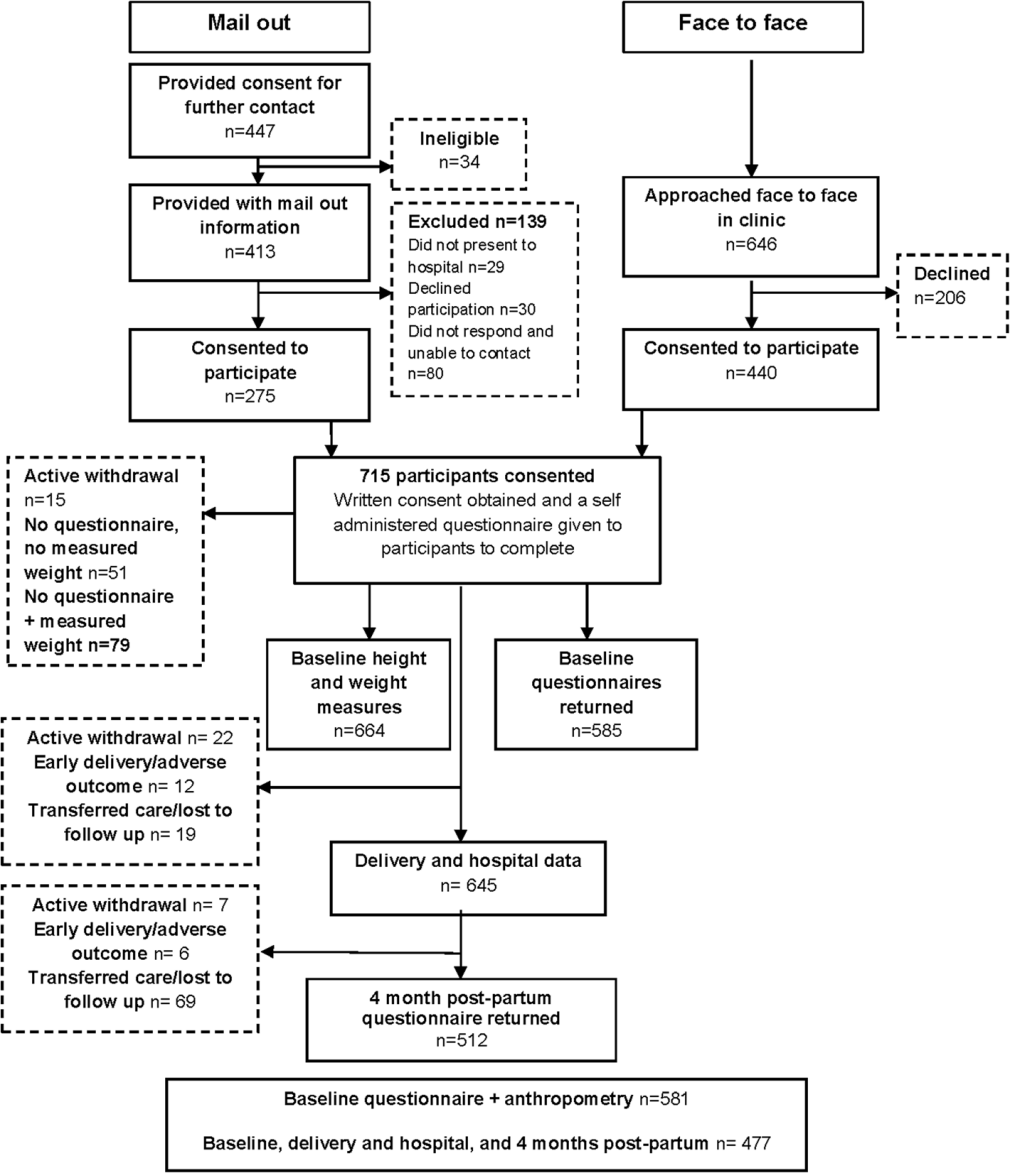
Progression of participants through the *New Beginnings* study time points from recruitment through to 4 month post-partum follow up

The present study is based on secondary analysis of data from 477 mothers enrolled in the *New Beginnings: Healthy Mothers and Babies* study, details of which have been previously described [10]. Flow of participants through the study is shown in Fig. 1. Using a consecutive sampling approach women were contacted via mailed registration packages or were approached in person in the antenatal clinic between August 2010 and January 2011 at the Royal Brisbane and Women’s Hospital. Eligibility criteria were: 18 years of age or older, without pre-existing type 1 or 2 diabetes, and with sufficient English language skills to complete questionnaires. Of the 715 women who provided written consent to participate, 664 provided baseline anthropometric data and 581 provided both baseline anthropometric and survey data.

The initial sample (*N* = 581) who provided data at baseline (~ 16 weeks gestation) was broadly representative of the Queensland obstetric population with respect to age, marital status, ethnicity, parity and anthropometric characteristics [12]. The present study was predominantly based on retrospective data on infant feeding in the first month after delivery that was self-reported by participants at approximately 4 months postpartum. Comparisons between participants included (*N* = 477) and those not included in the present analysis due to loss to follow-up or missing data were based on baseline data available from 581 participants on key demographic variables. Women included were older (M = 30 ± SD = 5 vs M = 28 ± SD = 6), more likely to be married or in a defacto relationship (96% vs 87%), be born in Australia (72% vs 56%), be multiparous (40% vs 25%) and have a university level education (47% vs 38%).

### Measures

#### Demographic and perinatal characteristics

At baseline (16 weeks gestation) data on maternal age (years), education (university vs. no university), parity (nulliparious vs. multiparous), marital status (married/de facto vs. other), country of birth (Australia vs. other), and possession of a health care card (yes vs. no) were collected via a self-reported questionnaire. Method of delivery (vaginal [including assisted] vs. caesarean section), infant gestational age, gender and birth weight were collected from hospital perinatal records. Information on postpartum depressive symptomatology (Edinburgh Postnatal Depression Scale [13]) and smoking status (smoker vs. non-smoker) were collected via self-report at 4 months postpartum.

#### Anthropometry

Pre-pregnancy BMI (kg/m^2^) was calculated using self-reported pre-pregnancy weight and measured height collected at 16 weeks gestation. World Health Organization guidelines were used to classify participants as: underweight (< 18.5 kg/m^2^), normal weight (18.5 to 24.9 kg/m^2^), pre-obese (25.0 to 29.9 kg/m^2^) or obese (≥30 kg/m^2^) [14]. Only a small percentage of women in the current sample were categorized as underweight (4.5%) and obese (10%) prior to pregnancy. Therefore women were allocated to either the (i) “non-overweight” pre-pregnancy group (includes underweight and normal weight status categories; mean BMI = 21 ± SD = 2), or (ii) “overweight” pre-pregnancy group (includes overweight and obese weight status categories; mean BMI = 29 ± SD = 5). Binary categorization of weight status is common in body weight literature [15, 16].

#### Breastfeeding in the first month postpartsum

Women were asked to indicate whether they experienced any of nine specified breastfeeding problems (plus an “other problems” category) during the first month after delivery and how each problem had impacted on breastfeeding their baby. The following five response options were available: (1) did not have this; (2) had this but continued breastfeeding without help; (3) had this and continued breastfeeding with professional advice; (4) had this and gave baby formula but continued breastfeeding, and (5) had this and was unable to keep breastfeeding. For the purposes of this analysis responses were recoded as “did not have this problem”, “had problem but continued exclusive breastfeeding”, and “had this problem and gave formula (either with or without continued breastfeeding)”. Women who did **not** breastfeed in the first month could skip this section of the questionnaire by checking the response box labelled “This question does not apply to me, I did not breastfeed”.

#### Use of infant formula in the first month postpartum

Sixteen reasons for the use of infant formula in the first month after delivery were adapted from the study by Li et al. [7] Women were asked to rate how important each reason was to their decision to give formula on a 4 point scale: (1) strongly disagree; (2) somewhat disagree; (3) somewhat agree, and (4) strongly agree. As per Li et al. [7] responses were dichotomized as “agree” or “disagree”. Women who did **not** give formula in the first month could skip this section of the questionnaire by checking the response box labelled “This question does not apply to me, my baby was only fed with breastmilk”.

### Statistical analyses

All statistical analyses were conducted using SPSS (Version 22; SPSS Inc., Chicago, IL, USA). Differences between non-overweight and overweight women on demographic and perinatal variables (see Table 1) were assessed using independent samples t-tests or Pearson chi-square tests (2-sided). Comparison between non-overweight and overweight women in terms of (i) whether they reported experiencing each of the specified breastfeeding problems (yes vs. no) and (ii) whether they gave formula (yes vs. no) in response to each of the specified breastfeeding problems were conducted using logistic binary regression adjusting for a priori selected covariates (age, education, country of birth, parity (1 child vs > 1 child) and method of delivery (vaginal vs caesarean section)). The first (i) set of analyses excluded women who reported that they did not breastfeed their infant in the first month after delivery and the second (ii) set of analyses included only those women who reported experiencing the specific breastfeeding problem. Finally, using only data from women who had given formula in the first month, comparisons between non-overweight and overweight women in terms of whether they agreed (yes vs. no) that specified reasons were important in their decision to give infant formula were conducted using logistic binary regression adjusting for a priori selected covariates indicated above. Comparisons of non-overweight and overweight groups were not conducted if there were less than 5 cases per cell.

**Table 1:** Demographic, perinatal and breastfeeding characteristics of women and their infants included in the study

Characteristic	Total (*N* = 477)	Non-overweight (*n* = 315)	Overweight (*n* = 162)	*P* value ^a^
	% (n) or M ± SD			
Education (university degree)	47 (224)	52 (163)	38 (61)	.003
Age (years)	30 **±** 5	30 **±** 5	30 **±** 5	.83
Parity (> 1 child)	42 (197)	41 (128)	43 (69)	.74
Marital status (married/de facto)	96 (457)	96 (301)	96 (156)	.70
Country of birth (Australia)	72 (341)	68 (212)	80 (129)	.005
Health care card (yes) ^b^	15 (71)	14 (44)	17 (27)	.43
Edinburgh Postnatal Depression Scale [13] Score ^c^	6 **±** 4	6 **±** 4	6 **±** 5	.29
Smoking status (non-smoker) ^d^	93 (444)	94 (297)	91 (147)	.19
Infant gender (boy)	52 (244)	52 (164)	50 (80)	.72
Infant birth weight (g)	3457 **±** 520	3448 **±** 450	3473 **±** 637	.66
Gestational age (weeks)	40 **±** 1	40 **±** 1	40 **±** 1	.94
Vaginal delivery (including assisted)	70 (333)	76 (238)	59 (95)	<.001

Non-overweight group: BMI < 25 kg/m^2^; Overweight group: BMI ≥25 kg/m^2^

^a^Difference (*P* value) for Pearson Chi-Square statistic (categorical variable) or Independent samples t-test (continuous variable)

^b^A health care card is issued by the Australian government to eligible people with low incomes. This card entitles the holder to health services and medicines at reduced cost

^c^Self-reported at 4 months postpartum; score range from 0 to 30

^d^Self-reported at 4 months postpartum

## Results

Characteristics of the 477 women who provided data on feeding their baby in the first month after delivery are shown in Table 1. Non-overweight and overweight women were similar on the majority of variables with a few notable exceptions. Overweight women were less likely to have a university level of education (*P* = .003), more likely to be born in Australia (*P* = .005) and less likely to have had a vaginal (including assisted) delivery (*P* < .001) compared to non-overweight women. Overall 96.8% of the sample initiated breastfeeding and there was no difference between non-overweight and overweight women: 12 out of 315 (3.8%) non-overweight women and 3 out of 162 (1.9%) overweight women reported that they never breastfed.

### Self-reported breastfeeding problems

Ninety percent of women who did initiate breastfeeding (416 out of 462) reported at least one breastfeeding problem in the first month. The average number of breastfeeding problems experienced during the first month was comparable between non-overweight (M = 2.7 ± SD = 1.6) and overweight (M = 2.6 ± SD = 1.5) women, *P* = .40. There were no significant differences between the proportion of non-overweight and overweight women who experienced these problems, after adjusting for selected covariates. The proportion of women experiencing breastfeeding problems is presented in Table 2 (see Appendix for list of “other” breastfeeding problems). The proportion of mothers who gave infant formula (either in combination with breastfeeding or alone) in response to specified breastfeeding problems is shown in Table 3. Again, there were no significant differences between non-overweight and overweight women after adjusting for selected covariates except that more overweight than non-overweight mothers reported giving formula for ‘other’ reasons (OR = 12.68, 95% CI = (1.46, 109.93, *P* = .021.

**Table 2:** Logistic regression analyses comparing proportions of non-overweight and overweight women who reported breastfeeding problems in the first month postpartum

Breastfeeding Problem	Total (*n* = 462)	Non-overweight (*n* = 303)	Overweight (*n* = 159)	Unadjusted	Adjusted ^a^	
	% (n) reporting the problem			*P* value	OR (95% CI)	*P* value
Sore or cracked nipples	61 (283)	66 (197)	55 (86)	.027	0.66 (0.44, 0.99)	.046
Latching or attachment	53 (245)	56 (169)	48 (76)	.12	0.73 (0.49, 1.11)	.14
Difficulties positioning	37 (172)	39 (115)	37 (57)	.69	1.09 (0.71, 1.68)	.69
Too much milk	26 (121)	29 (86)	23 (35)	.15	0.76 (0.47, 1.22)	.25
Not enough milk	25 (114)	23 (67)	32 (47)	.071	1.48 (0.93, 2.37)	.10
Delay in milk coming in	21 (99)	20 (61)	25 (38)	.33	1.22 (0.74, 2.01)	.43
Mastitis	15 (71)	16 (48)	15 (23)	.72	1.08 (0.61, 1.90)	.80
Baby refused breast	11 (49)	10 (30)	12 (19)	.48	1.30 (0.68, 2.45)	.43
Baby tongue tie	8 (36)	7 (22)	9 (14)	.54	1.52 (0.72, 3.18)	.27
Other ^b^	9 (42)	9 (27)	10 (15)	.84	1.13 (0.57, 2.27)	.72

Non-overweight: BMI < 25 kg/m^2^; Overweight: BMI ≥25 kg/m^2^

n.b. Analysis based on mothers who initiated breastfeeding (462 out of 477); breastfeeding problems are not mutually exclusive

^a^Adjusted for maternal age, education, parity, birth country, family health care card and mode of delivery. N value for adjusted analyses = 454 due to some missing data on covariates

^b^see Appendix for list of “other” breastfeeding problems

**Table 3:** Logistic regression analyses comparing proportions of non-overweight and overweight women who gave formula milk in response to self-reported breastfeeding problems in the first month postpartum

Breastfeeding Problem	Total	Non-overweight	Overweight	Unadjusted	Adjusted ^b^	
	% of women reporting problem who gave formula in response (n/total) ^a^			*P* value	OR (95% CI)	*P* value
Sore or cracked nipples	10 (28/283)	11 (22/197)	7 (6/86)	.28	0.47 (0.16, 1.36)	.17
Latching or attachment	17 (42/245)	14 (23/169)	25 (19/76)	.029	1.64 (0.76, 3.54)	.21
Difficulties positioning	12 (20/172)	10 (11/115)	16 (9/57)	.23	1.66 (0.54, 5.13)	.38
Too much milk	3 (4/121)	2 (2/86)	6 (2/35)	–	–	–
Not enough milk	71 (81/114)	70 (47/67)	72 (34/47)	.80	0.72 (0.28, 1.83)	.49
Delay in milk coming in	51 (50/99)	48 (29/61)	55 (21/38)	.46	1.35 (0.51, 3.62)	.55
Mastitis	16 (11/71)	17 (8/48)	13 (3/23)	–	–	–
Baby refused breast	37 (18/49)	30 (9/30)	47 (9/19)	.22	1.67 (0.36, 7.77)	.52
Baby tongue tie	22 (8/36)	23 (5/22)	21 (3/14)	–	–	–
Other ^b^	33 (14/42)	22 (6/27)	53 (8/15)	.04	12.68 (1.46, 109.93)	.021

Non-overweight: BMI < 25 kg/m^2^; Overweight: BMI ≥25 kg/m^2^

n.b. Analyses based on participants who reported each specified breastfeeding problem (n values given in table); breastfeeding problems are not mutually exclusive; logistic regression analysis not conducted if < 5 cases in either weight status group who reported giving formula in response to a breastfeeding problem

^a^Based only on those participants who reported that they experienced the specified problem

^b^Adjusted for maternal age, education, parity, birth country, family health care card and mode of delivery. N value for adjusted analyses ~ 7% lower than unadjusted analyses due to some missing data on covariates

### Reasons for giving infant formula

A greater proportion of overweight women (49%) relative to non-overweight women (32%) used formula during the first month (*P* < .001). This difference remained significant after adjusting for selected covariates (adjusted OR = 1.83, 95% CI = 1.21, 2.78, *P* = .004). Reasons for use of infant formula are listed in Table 4 in order of the proportion of women who agreed that the reason was important. Overweight women were more likely than non-overweight women to agree that an important reason for why they used formula in the first month was that it was just as good as breastfeeding (adjusted OR = 2.78, 95% CI = 1.31, 5.91). Non-overweight women were more likely than overweight women to agree that health professional advice was an important reason for why they used formula in the first month (adjusted OR = .40, 95% CI = .19, .85).

**Table 4:** Logistic regression analyses comparing proportions of non-overweight and overweight women who agreed that specific reasons were important in their decision to use infant formula in the first month postpartum

Important Reason for use of Infant Formula	Total (*n* = 177)	Non-overweight (*n* = 98)	Overweight (*n* = 79)	Unadjusted	Adjusted ^a^	
	% (n) who “agreed” reason was important			*P* value	OR (95% CI)	*P* value
Did not have enough milk	49 (88)	48 (48)	51 (40)	.73	1.05 (0.54, 2.16)	.88
Baby didn’t put on enough or lost weight	34 (61)	31 (31)	38 (30)	.35	1.15 (0.56, 2.37)	.71
Health professional advice	31 (56)	37 (37)	24 (19)	.064	0.36 (1.63, .81)	.013
Formula just as good as breastfeeding	30 (54)	22 (22)	41 (32)	.008	2.28 (1.06, 4.92)	.036
Tried breastfeeding before and didn’t like it	16 (29)	15 (15)	18 (14)	.60	1.06 (0.42, 2.70)	.89
Didn’t feel comfortable feeding in public	16 (29)	15 (15)	18 (14)	.60	1.80 (0.71, 4.56)	.22
Mum sick or on medications	15 (27)	14 (14)	17 (13)	.65	1.16 (0.45, 2.95)	.76
Baby sick or preterm	14 (25)	11 (11)	18 (14)	.22	1.40 (0.54, 3.68)	.49
Needed someone else to feed baby	13 (23)	15 (15)	10 (8)	.32	0.57 (0.19, 1.71)	.32
I wanted to leave baby for hours	13 (23)	15 (15)	10 (8)	.38	0.58 (0.19, 1.74)	.33
Someone else wanted to feed baby	11 (19)	14 (14)	6 (5)	.10	0.37 (0.11, 1.24)	.11
Breastfeeding too inconvenient	7 (12)	5 (5)	9 (7)	.31	2.16 (0.60, 7.77)	.24
I had too many household duties	6 (11)	7 (7)	5 (4)	.58	0.83 (0.19, 3.52)	.80
Wanted my body back	4 (7)	2 (2)	6 (5)	–	–	–
Wanted to go on diet	1 (2)	2 (2)	0 (0)	–	–	–
Wanted to smoke or drink alcohol	1 (1)	1 (1)	0 (0)	–	–	–

Non-overweight: BMI < 25 kg/m^2^; Overweight: BMI ≥25 kg/m^2^

n.b. Analyses based on participants who gave formula during the first month postpartum (177 out of 477); reasons for use of infant formula are not mutually exclusive; analysis not conducted if < 5 cases in either weight status group who “agreed” reason was important

^a^Adjusted for birth country (mother), mode of delivery and education. N value for adjusted analyses = 172 due to some missing data on covariates

## Discussion

The aim of the present study was to examine whether women who were overweight prior to pregnancy would be more likely to self-report a range of breastfeeding problems and be more likely to use formula in response to these problems during the first month postpartum, even after adjusting for covariates. A second aim was to examine whether overweight and non-overweight women differed in their reasons for use of infant formula in the first month postpartum. In this sample it was previously reported that women who entered their pregnancy overweight were more likely to have ceased exclusive breastfeeding by 4 months [11] and here we show that overweight women were also more likely to report having used formula in the first month compared to non-overweight women. Although almost all mothers reported that they had experienced at least one of a range of specified breastfeeding problems during the first month postpartum, there were no statistically significant differences according to weight status with covariate adjustment. However, overweight mothers were more likely than non-overweight mothers to use infant formula in response to unspecified (i.e., ‘other’) breastfeeding problems and were more like to use infant formula during the first month because they believed it was just as good as breastfeeding. Self-reported problems with milk supply did not differ between weight groups but was the most commonly reported reason for use of infant formula.

No differences in prevalence of a list of specified breastfeeding problems or use of formula to manage these problems was found between weight status groups in the present study, however more overweight than non-overweight women had used infant formula in the first month, and significantly more overweight women than non-overweight women had ceased breastfeeding by the time of the survey (approximately 4 months postpartum). [11] Over half of overweight women who reported an unspecified (‘other’) breastfeeding problem used formula in response. Further exploration of these problems (in particular thrush and baby being a ‘lazy feeder’) is needed to better understand this finding. Examination of reasons for formula use in the first month provides some insight into a possible reason for the different outcomes between weight status groups. Almost double the proportion of overweight women than non-overweight women who used infant formula in the first month agreed that an important reason for using formula was that it was as good as breastmilk. This finding is particularly concerning given that children of overweight women have higher obesity risk, [5] which may be compounded by not breastfeeding [1]. Further qualitative research may assist in understanding the reasoning behind these issues and how best to communicate the range of health, social and economic benefits of breastfeeding and risks associated with formula feeding to women who may be entering pregnancy overweight or obese.

An unexpected finding was that there was no difference in the proportion of overweight and non-overweight women who reported having “not enough milk” in the first month and who used infant formula in response. These findings are inconsistent with conclusions of a recent review paper [3]. However longer-term impacts on breastfeeding (such as early cessation) were not assessed here and potentially issues with milk supply (real or perceived) may have differential impacts on breastfeeding outcomes for overweight compared to non-overweight women in the longer-term. Nevertheless, we did show that perceived insufficient milk supply was a common problem affecting over two thirds of breastfeeding mothers during the first month; with almost half of these women using infant formula in response. It is important to note that the data reflect mothers’ *perceptions* rather than actual adequacy of supply to meet infant energy requirements; it has been estimated that only a small minority of women (~ 5%) have physiologic insufficient milk supply [17]. Previous work has indicated that perceptions of insufficient milk are consistently associated with low maternal self-efficacy [18, 19]. Thus, antenatal and postnatal breastfeeding guidance and support needs to consider how concerns about milk may be ameliorated through increasing women’s breastfeeding self-efficacy.

The current findings also did not support the notion that overweight women were more likely than non-overweight women to give infant formula because they felt self-conscious about breastfeeding in public [8]. However feeling uncomfortable feeding in public was a salient concern for a subset of non-overweight (15%) and overweight mothers (18%) who gave formula in the first month. This concern is a potential barrier to successful breastfeeding that may reflect social and cultural perspectives on the acceptability of women breastfeeding. On a societal level, this barrier to breastfeeding can be addressed through legislation and policy that both protects and promotes the rights of women to breastfeed in public places. On an individual level, health professionals could discuss with new (or expectant) mothers that their right to breastfeed in public is protected by law. It may also be helpful to discuss potential strategies to manage feeling self-conscious breastfeeding in public such as the use of dedicated breastfeeding rooms in shopping centres.

The findings described here need to be considered within the cultural context of Australia and the specific limitations of the study. Relative to other Western countries Australia has high rates of breastfeeding initiation however, the rate of initiation in this sample (96.8%) appears higher than nationally representative data (breastfeeding initiation = 87.8% based on data from a 2004–2005 health survey) [20]. Furthermore, due to attrition between baseline and follow-up the final sample was not representative of the obstetric population of interest and was over-represented by English-speaking women who were older, married and university educated. A major limitation of the study is the reliance on self-report data which may be subject to social desirability bias. Additionally, the use of retrospective self-report measures of breastfeeding problems in the first month and pre-pregnancy weight may also be subject to recall bias. Combining underweight with normal weight and pre-obese with obese to form the “non-overweight” and “overweight” groups, respectively, was necessary for statistical reasons (i.e., power) but may have obscured differences unique to women with very low or very high BMIs. An additional limitation was that some of the analyses reported here involved a smaller subset of the sample (i.e., only those women who had used formula in the first month) and thus potential groups differences may have failed to reach statistical significance due to insufficient power.

## Conclusions

The current study adds to previous literature by comparing prevalence of specific breastfeeding-related problems and reasons for use of infant formula between non-overweight and overweight women. Overall the data suggest that the experience of breastfeeding problems in the first month is very common for women regardless of pre-pregnancy weight status. Concerns about milk supply emerged as the main problem associated with use of infant formula in the first month in both weight status groups. Overweight women were more likely than non-overweight women to use infant formula because they believed that it was “as good as breastfeeding”. Further research into why overweight women in particular may hold this belief is warranted and may clarify expectations or attitudes of healthcare staff and level of support for breastfeeding that is offered to overweight compared the non-overweight women. Nevertheless, prenatal and antenatal breastfeeding support and education that addresses concerns around milk supply are likely to benefit all mothers, regardless of weight status.

## Appendix

**Table 5:** “Other” breastfeeding problems reported during first month after delivery

“Other” breastfeeding problems	n
Thrush (oral or nipple)	11
Sleepy baby/Lazy sucker	6
Reflux	5
Maternal ill health/stress	3
Baby demanded frequent feeds (every 1-2 h)	2
Jaundice	2
Blocked milk ducts	2
Previous breast surgery	1
Flat nipples	1
Laryngomalacia (congenital abnormality of the laryngeal cartilage)	1
Baby ill health	1
Twins	1
Baby mouth to small	1
Baby swallowing air	1
Sensitive nipples	1
Hypoplasia (tubular shaped breasts)	1
Mother concerned that baby fed too quickly	1
Fast flow	1

n.b. only one reason reported per participant (*n* = 42)

## Abbreviation

BMI: Body mass index
